# Arginine deficiency augments inflammatory mediator production by airway epithelial cells in vitro

**DOI:** 10.1186/1465-9921-10-62

**Published:** 2009-07-03

**Authors:** Xiao-Yun Fan, Arjen van den Berg, Mieke Snoek, Laurens G van der Flier, Barbara Smids, Henk M Jansen, Rong-Yu Liu, René Lutter

**Affiliations:** 1 Department of Pulmonology, The Geriatric Institute of Anhui, The First Affiliated Hospital of Anhui Medical University, Hefei, Anhui 230022, PR China; 2 Department of Pulmonology Academic Medical Centre, University of Amsterdam, Amsterdam, the Netherlands; 3 Department of Experimental Immunology, Academic Medical Centre, University of Amsterdam, Amsterdam, the Netherlands

## Abstract

**Background:**

Previously we showed that reduced availability of the essential amino acid tryptophan per se attenuates post-transcriptional control of interleukin (IL)-6 and IL-8 leading to hyperresponsive production of these inflammatory mediators by airway epithelial cells. Availability of the non-essential amino acid arginine in the inflamed airway mucosa of patients with asthma is reduced markedly, but it is not known whether this can also lead to an exaggerated production of IL-6 and IL-8.

**Methods:**

IL-6 and IL-8 were determined by ELISA in culture supernatants of NCI-H292 airway epithelial-like cells and normal bronchial epithelial (NHBE) cells that were exposed to TNF-α, LPS or no stimulus, in medium with or without arginine. Arginine deficiency may also result from exposure to poly-L-arginine or major basic protein (MBP), which can block arginine uptake. Epithelial cells were exposed to these polycationic proteins and L-^14^C-arginine uptake was assessed as well as IL-6 and IL-8 production. To determine the mode of action, IL-6 and IL-8 mRNA profiles over time were assessed as were gene transcription and post-transcriptional mRNA degradation.

**Results:**

For both NCI-H292 and NHBE cells, low arginine concentrations enhanced basal epithelial IL-6 and IL-8 production and synergized with TNF-α-induced IL-6 and IL-8 production. Poly-L-arginine enhanced the stimulus-induced IL-6 and IL-8 production, however, blocking arginine uptake and the enhanced IL-6 and IL-8 production appeared unrelated. The exaggerated IL-6 and IL-8 production due to arginine deficiency and to poly-L-arginine depend on a post-transcriptional and a transcriptional process, respectively.

**Conclusion:**

We conclude that both reduced arginine availability per se and the presence of polycationic proteins may promote airway inflammation by enhanced pro-inflammatory mediator production in airway epithelial cells, but due to distinct mechanisms.

## Background

Asthma is an inflammatory airway disease characterized by intermittent and variable degrees of airway obstruction and bronchial hyperresponsiveness [1-3]. There are several studies that point to enhanced arginine catabolism in airways of patients with asthma, which may lead to reduced local bioavailability of arginine. Arginine is catabolised to the potent smooth muscle cell relaxing component nitric oxide (NO) and L-citrulline by constitutive nitric oxide synthases (neuronal NOS and endothelial NOS) and the inducible nitric oxide synthase (iNOS). Enhanced NO levels are found in exhaled air of patients with asthma, which reflects particularly the enhanced expression of iNOS in airway epithelial cells [4,5]. Furthermore, a study by Kochansky and colleagues [6] showed enhanced arginase activity in sputum from patients with asthma. This was independently corroborated in a study by Zimmerman et al. [7] showing abundant expression of arginase-I at the mRNA and protein level in airway epithelial cells from patients with asthma. Increased enzymatic activity was confirmed by showing reduced arginine levels in serum samples from exacerbating asthmatics [8], suggestive of a larger depletion of arginine locally in the airways.

Our previous studies into the regulation of the production of inflammatory mediators, exemplified by key pro-inflammatory interleukin(IL)-6 and IL-8, by airway epithelial cells have shown that reduced levels of the essential amino acid tryptophan, which affects cellular metabolism, led to exaggerated production of these mediators by post-transcriptional mechanisms [9]. It is unknown whether reduced levels of arginine per se give rise to similar exaggerated mediator responses. Arginine is a non-essential amino acid, acquired by eukaryotic cells by synthesis in the Krebs urea cycle and uptake via the cationic amino acid transporters (CAT; [10-14]). CATs are blocked by polycations like major basic protein (MBP). MBP is present in airways of asthmatics and thus may also lead to reduced arginine availability to cells, even in the presence of extracellular arginine [15]. We were interested to assess whether reduced extracellular arginine levels as well as blocking arginine uptake by polycations would influence the epithelial production of the inflammatory mediators IL-6 and IL-8.

## Methods

### Cell culture and experimental set up

NCI-H292 cells (CRL 1848; American Type Culture Collection [ATCC], Manassas, VA) were cultured and propagated in RPMI 1640 medium with 10% fetal calf serum (FCS), 100 U/ml penicillin, 100 μg/ml streptomycin and 1.2 mM L-glutamine (H292 medium). For some experiments, cells were incubated with minimum essential medium Eagle (MEM), modified with Earle's salts, 2 g/L sodium bicarbonate without L-glutamine and L-arginine (ICN Biomedicals, Zoetermeer, the Netherlands) supplemented with L-glutamine, penicillin and streptomycin as indicated above. For mediator release, 3 × 10^5 ^cells were plated and cultured overnight in 500 μl in 24-well plates. For isolation of mRNA and nuclear extracts, 15 × 10^5 ^cells were plated and cultured overnight in 2.5 ml in 6-well plates. Cells were washed and then exposed in fresh medium for 20 hours to doses of pathophysiologically relevant stimuli, i.e. recombinant human TNF-α (R&D systems, Minneapolis, MN), LPS (Sigma Chemical Co., St. Louis, MO) or recombinant human IFN-γ (Roche Molecular Biochemicals) up to 5 ng/ml, 5 μg/ml or 100 U/ml, respectively. When indicated arginine, poly-L-arginine, heparin (Merck; Nottingham, UK) or N-Nitro-arginine Methyl Ester (L-NAME; Sigma Chemical Co.) were added. For a number of experiments we used primary bronchial epithelial cells (NHBE; Lonza Benelux BV). NHBE cells were cultured and propagated as recommended by the supplier, and cells were used between passage 3 and 5. For cytokine production, 0.2 × 10^5 ^cells were plated in 48-well plates, and before exposure to stimuli, cells were washed with PBS and incubated with MEM plus or minus arginine. Cell cultures were screened on a routine basis for Mycoplasma by PCR and were found negative. In all experiments t = 0 is collected directly after all cells were exposed to the indicated experimental conditions.

### L-^14^C-arginine uptake

NCI-H292 cells (6 × 10^4 ^cells) were plated in 100 μl H292 medium per well in a 96-well plate. After overnight incubation, cells were washed with 100 μl Hank's balanced salt solution (HBSS) without arginine and then exposed to poly-L-arginine or major basic protein (MBP) in 100 μl HBSS. Then, 10 μl per well of 100 μM L-^14^C-arginine (Amersham Biosciences, UK) was added. After 30 minutes, cells were washed 3 times with 100 μl HBSS and lysed for 5 minutes in a final 0.1% (v/v) Triton X-100 in PBS. After the addition of 100 μl scintillation fluid, radioactivity was counted.

### Measurement of IL-6 or IL-8 protein and mRNA

IL-6 and IL-8 in culture supernatants were measured by sandwich ELISA, as described before [16,17]. Total RNA was extracted with Trizol (Invitrogen, Paisley, UK) and IL-6, IL-8 and GAPDH mRNA were determined by dotblotting and hybridization with specific ^32^P-labeled probes for IL-6, IL-8 and GAPDH as described [18,19]. Previous studies had confirmed that dotblotting yielded similar results as Northern blotting [9,20,21]. Blots were quantified using a phosphorimager and variable loading was corrected by expressing mRNA levels relative to that of the housekeeping gene GAPDH. mRNA decay was determined 2 hours after stimulation by co-incubation with 5 μg/ml of the transcriptional blocker actinomycin-D (Sigma Chemical Co.). At time zero and after an additional 40 and 80 minutes, remaining IL-6 and IL-8 mRNA were determined, as described above.

### Transcriptional activity

Transcriptional activity was assessed by measuring a reporter protein under strict control of the IL-6 or IL-8 promoter. To that end, NCI-H292 cells were grown to 70% confluence in 6-well plates and transfected with 5 μg of chloramphenicol acetyltransferase (CAT) reporter construct driven by the wild-type IL-8 [22] or IL-6 promoter [23], as described [18-20]. Cells were stimulated as indicated, 24 h after transfection and 18 h before cell lysis. CAT production was measured by CAT ELISA (Roche Diagnostics, Mannheim, Germany) according to the manufacturer's protocol and all data were normalized for total protein content.

### Statistics

All parameters and comparisons were performed using SPSS 11.5 software. Data are expressed as means ± standard error of the mean (SEM). The paired or Independent-Samples t-test and Pearson Correlation were used when appropriate to evaluate statistical significance. For multiple comparisons we used one-way ANOVA, followed by least significance difference when equal variances are assumed or Dunnett's T_3 _when no equal variances are assumed, and the multi-regression model method. Differences were considered significant at *P *≤ 0.05.

## Results

### Reduced levels of arginine enhanced IL-6 and IL-8 production by airway epithelial cells

**Figure 1:**
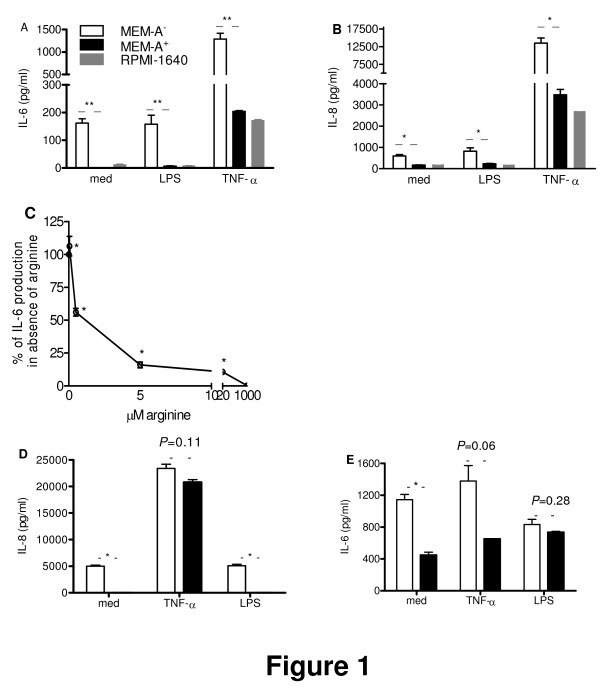
**Enhanced production of IL-6 and IL-8 by airway epithelial cells as a function of arginine**. NCI-H292 cells were plated and, after overnight culture, exposed for 24 h to fresh medium (RPMI-1640 plus L-glutamine, MEM plus L-glutamine and arginine (MEM-A^+^) and MEM with L-glutamine only (MEM-A^-^)) without and with stimuli (LPS: 5 μg/ml; TNF-α: 5 ng/ml). Typical experiment (**A**; IL-6 production; **B**: IL-8 production) is shown out of three experiments (triplicate samples; mean ± SEM, *P < 0.05; **P ≤ 0.001). Error bars may be contained within the bar. **C**) IL-6 production as a function of arginine concentration relative to IL-6 production in the absence of arginine (*P ≤ 0.019). **D**) IL-8 production by NHBE cells in MEM-A^+ ^and MEM-A^- ^medium (*P ≤ 0.003). **E**) IL-6 production by NHBE cells after 8 h. in MEM-A^+ ^and MEM-A^- ^medium (*P ≤ 0.012).

The normal cell culture medium for NCI-H292 cells, RPMI-1640 medium (containing 1 mM arginine) supplemented with L-glutamine, penicillin, streptomycin and 10% FCS (with < 100 μM arginine), thus contains between 0.9 to 1 mM arginine. Initial studies showed that NCI-H292 cells can be maintained in RPMI-1640 medium without FCS for a week without evident morphological and functional (IL-6 and IL-8 production) effects on the cells, and further, that RPMI-1640 medium can be replaced with MEM, which allowed us to control extracellular arginine levels. Figures 1A and 1B show that the basal as well as LPS- and TNF-α-stimulated IL-6 and IL-8 production was similar for NCI-H292 cells maintained in either RPMI-1640 medium or MEM supplemented with L-glutamine and arginine (MEM-A^+^). LPS, however, acted as a poor stimulus in the absence of FCS. The IL-6 and IL-8 production by cells maintained in MEM without arginine (MEM-A^-^), as compared to that of cells in medium containing 1 mM arginine (MEM-A^+^) or in RPMI-1640 medium was markedly increased (IL-6: *P *< 0.001, *P *= 0.001 and *P *< 0.001; IL-8: *P *= 0.048, *P *= 0.005 and *P *= 0.037 for non-stimulated, LPS- and TNF-α-stimulated cells, respectively). At 20 μM and 5 μM arginine, IL-6 and IL-8 (not shown) production were still significantly enhanced over that at 1 mM arginine (Figure 1C). In fact, between 0 to 1 mM arginine, the concentration of arginine inversely correlated with IL-6 production (r = -0.513; p = 0.03). We did not find a similar correlation for IL-8 production between 0 to 1 mM arginine.

Primary bronchial epithelial cells (NHBE) could be kept in MEM medium for a limited period only as cell morphology changed and cells detached over time. NHBE cells in MEM-A^- ^for 21 h displayed enhanced production of IL-8 as compared to NHBE cells in MEM-A^+^, in the absence of a stimulus (*P *= 0.001), with LPS (*P *= 0.003), but not with TNF-α (*P *= 0.11; Figure 1D). Similar findings were obtained for IL-6 (Figure 1E). LPS, however, proved a poor stimulus of IL-6 and IL-8 production by NHBE cells at these culture conditions, i.e. in the absence of FCS.

### Hyperresponsive IL-6 and IL-8 production by reduced levels of arginine

**Figure 2:**
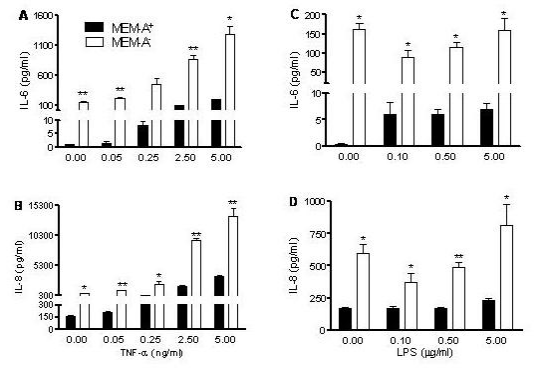
**Hyper-responsive IL-6 (A, C) and IL-8 (B, D) production by NCI-H292 cells maintained in MEM without arginine**. Cells were plated and, after overnight culture, exposed for 20 h to fresh medium (MEM plus L-glutamine and arginine (MEM-A^+^) or MEM with L-glutamine only (MEM-A^-^)) to a concentration range of TNF-α (**A**, **B**) and LPS (**C**, **D**). Typical experiment is shown out of three experiments (triplicate samples; mean ± SEM). *P < 0.05, **P < 0.01 and ***P < 0.001 as compared to MEM-A^+^.

In a previous study [9], in which the content of the essential amino acid tryptophan in culture medium was reduced, NCI-H292 cells displayed hyperresponsive IL-6 and IL-8 production to stimuli. When NCI-H292 cells were exposed to MEM medium with or without arginine we observed a hyperresponsive IL-6 and IL-8 production to TNF-α for cells in MEM-A^- ^(Figures 2A and 2B; dose dependency in MEM-A^- ^*r *= 0.945, *P *< 0.001; *r *= 0.967, *P *< 0.001, respectively). For cells in MEM-A^-^, no hyperresponsive IL-6 and IL-8 production to LPS was found, but merely an enhanced production in MEM-A^-^, similar to that found in the absence of a stimulus (Figures 2C and 2D; dose dependency in MEM-A^- ^*r *= 0.346, *P *= 0.271; *r *= 0.668, *P *= 0.018, respectively).

### Polycations enhance IL-6 and IL-8 production to LPS, independent of inhibition of arginine uptake

**Figure 3:**
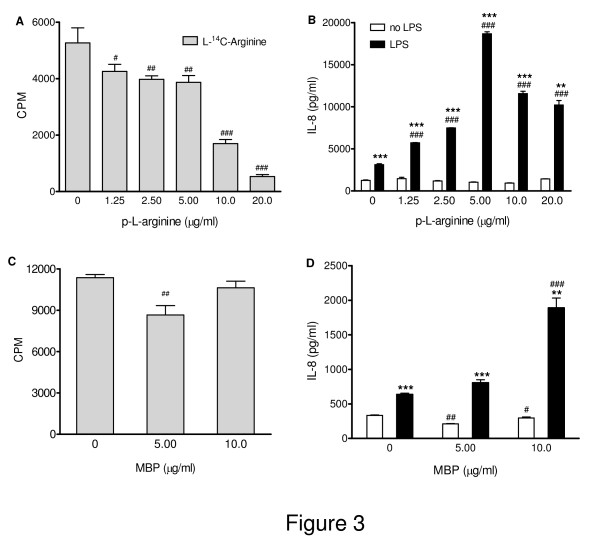
**The effect of polycations poly-L-arginine and major basic protein on L-^14^C-arginine uptake and LPS-induced IL-8 production by NCI-H292 cells**. Parallel cultures of NCI-H292 cells were exposed to different amounts of poly-L-arginine (**A**,**B**) or major basic protein (MBP; **C**,**D**). For arginine uptake (**A**, **C**) cells were washed with HBBS and L-^14^C-arginine was added for 30 min. (see Material and Methods). For IL-8 (**B**, **D**) and IL-6 (not shown) production the culture supernatants were collected after 20 h with no stimulus (white columns) or with 5 μg/ml LPS (black columns). Typical experiments are shown (triplicate samples; mean ± SEM) of 5 experiments with poly-L-arginine and 4 with MBP. *P < 0.05, **P < 0.01 and ***P < 0.001 for IL-8 production as compared to unstimulated cells; # *P *< 0.05, # # *P *< 0.01 and # # # *P *< 0.001 for uptake of L-^14^C-arginine as compared to that in the absence of poly-L-arginine or MBP.

**Figure 4:**
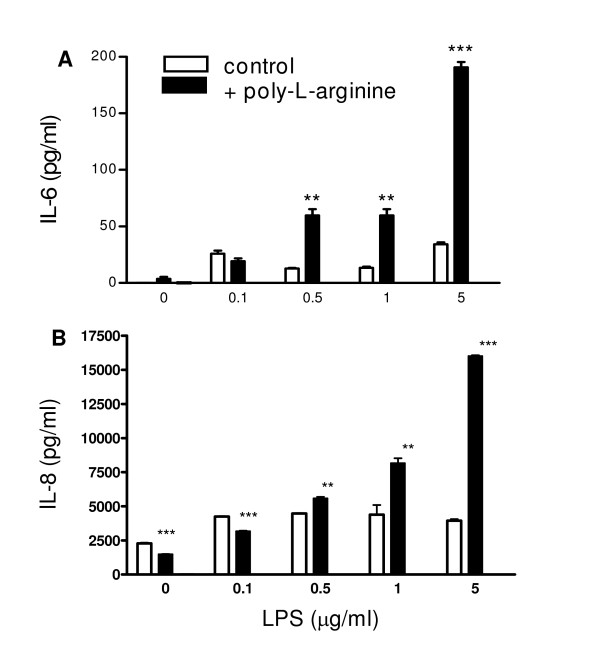
**Hyper-responsive IL-6 (A) and IL-8 (B) production by NCI-H292 cells exposed to poly-L-arginine**. Cells were exposed for 20 h to normal medium with or without 5 μg/ml poly-L-arginine and a concentration range of LPS. Typical experiment out of 5 is shown (triplicate samples; mean ± SEM). **P < 0.01 and ***P < 0.001 as compared to in the absence of poly-L-arginine.

Polycations like poly-L-arginine can inhibit cationic amino acid transporter proteins, among which those transporting arginine. We reasoned that the synergy in IL-6 and IL-8 production shown in the absence of extracellular arginine may also be accomplished by inhibiting arginine uptake with poly-L-arginine. Experiments to measure the effect of poly-L-arginine on L-^14^C-arginine uptake and on IL-6 and IL-8 production were performed in parallel, where L-^14^C-arginine uptake was assessed shortly after adding poly-L-arginine and IL-6 and IL-8 were measured in culture supernatants collected at 20 h after adding poly-L-arginine. Figure 3A shows that 10 and 20 μg/ml poly-L-arginine markedly reduced L-^14^C-arginine uptake (*P *< 0.001), and even at 1.25 to 5 μg/ml poly-L-arginine, arginine-uptake was inhibited significantly (*P *< 0.05 and *P *< 0.01, respectively). Forty μg/ml poly-L-arginine and higher appeared cytotoxic with cells detaching and thus was not tested further. Interestingly, basal IL-6 and IL-8 production were not affected by poly-L-arginine itself in contrast to the enhanced basal IL-6 and IL-8 production due to reduced arginine availability. At 1.25 μg/ml and higher, poly-L-arginine significantly potentiated LPS-induced IL-8 (*P *< 0.001) and IL-6 production (data not shown) with maximal synergy at 5 μg/ml of poly-L-arginine. Thus, inhibition of arginine uptake and the enhanced production of IL-8 and IL-6 did not correlate strictly. Heparin (10 μg/ml) fully inhibited the effect of poly-L-arginine on the LPS-induced IL-8 and IL-6 production, indicative of a role for the positive charges of poly-L-arginine. In contrast to the findings for LPS and to that seen after stimulation with TNF-α of NCI-H292 cells in MEM-A^-^, TNF-α did not synergize with poly-L-arginine in IL-6 and IL-8 production (data not shown). Exposure of NCI-H292 cells to LPS and poly-L-arginine resulted in a hyperresponsive IL-6 and IL-8 production (*P *= 0.015 and 0.046, respectively) (Figures 4A and 4B).

The eosinophilic polycation major basic protein (MBP) is present in airways secretions from patients with asthma. We next determined whether MBP, like poly-L-arginine, inhibited L-^14^C-arginine uptake and potentiated IL-6 and IL-8 production. MBP at 10 μg/ml largely increased the LPS-induced IL-8 (Figure 3B; *P *< 0.001) and IL-6 (data not shown) production, apparently independent of blocking uptake of L-^14^C-arginine (r = 0.06, *P *= 0.878) by NCI-H292 cells.

**Figure 5:**
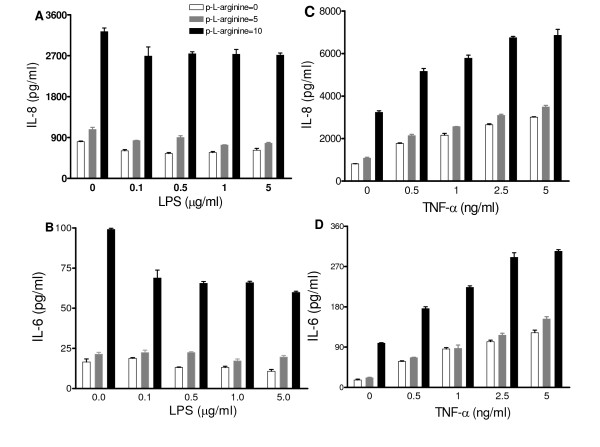
**Effect of poly-L-arginine on basal, LPS- and TNF-α-induced IL-6 (B, D) and IL-8 (A, C) production by NHBE cells**. Cells were exposed for 20 h to normal medium with or without 5 μg/ml poly-L-arginine and a concentration range of LPS or TNF-α. One out of two experiments is shown (triplicate samples; mean ± SEM). Poly-L-arginine significantly enhanced TNF-α-induced IL-6 and IL-8 production (P < 0.0001 and P < 0.0001, respectively).

In contrast to NCI-H292 cells, poly-L-arginine did not block arginine-uptake by NHBE cells (data not shown). Distinct from findings with the NCI-H292 cells, poly-L-arginine enhanced IL-6 and IL-8 production by NHBE cells in the absence of a stimulus (t = 13.161, *P *< 0.0001; t = 11.49, *P *< 0.0001; t = 10.045, *P *< 0.0001; t = 8.894, *P *< 0.0001, respectively for Figure 5A, B, C, D). TNF-α, but not LPS significantly synergized with poly-L-arginine in IL-6 and IL-8 production by NHBE cells, (multi regression model; t = 7.264, *P *< 0.0001 (5C); t = 6.002, *P *< 0.0001 (5D); t = -1.693, *P *= 0.098 (5A); t = -0.738, *P *= 0.464 (5B). Regression coefficients of curves with 10 μg/ml poly-L-arginine are steeper than those of 5 and 0 μg/ml poly-L-arginine).

### Enhanced IL-6 and IL-8 production by poly-L-arginine and reduced arginine levels: two distinct mechanisms

At suboptimal concentrations of arginine, iNOS has been shown to give rise to both NO and superoxide, yielding the potent oxidant peroxynitrite [11,24,25], that promotes tissue damage and thus inflammation [26]. Even though iNOS mRNA is expressed not or to a low extent in resting cells only, we assessed the contribution of NOS to IL-6 and IL-8 production by both NCI-H292 and NHBE cells using the non-specific NOS inhibitor L-NAME at various concentrations (0.01, 0.1 and 1 mM) and multiple conditions (no stimulus, LPS, TNF-α, poly-L-arginine and interferon gamma (IFN-γ)). Overall we found that L-NAME did not reduce IL-6 and IL-8 production unless cells were exposed to IFN-γ, which induced iNOS mRNA. NCI-H292 cells exposed to IFN-γ (100 U/ml) for 24 h were found to produce IL-6 and IL-8, which was inhibited for 50% by L-NAME.

**Figure 6:**
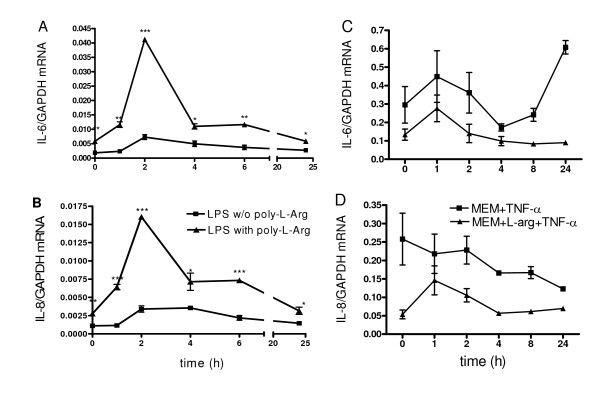
**IL-6 and IL-8 mRNA expression in NCI-H292 cells stimulated by LPS in the absence or presence of 5 μg/ml poly-L-arginine (A, B) or that of arginine (C, D)**. IL-6 and IL-8 mRNA levels were corrected for GAPDH mRNA. Typical experiments are shown (triplicate samples; mean ± SEM) of 4 (for poly-L-arginine) and 2 (for arginine) experiments for IL-6 as well as IL-8 mRNA. *P < 0.05, **P < 0.01 and ***P < 0.001 as compared to parallel samples in the absence of poly-L-arginine or presence of arginine.

To further explore the different mechanisms underlying the exaggerated IL-6 and IL-8 production by reduced arginine levels versus that by poly-L-arginine, we determined the IL-6 and IL-8 mRNA profiles in NCI-H292 cells over time for both conditions (Figure 6). In line with our previous findings, LPS increased IL-8 and IL-6 mRNA 2- to 3-fold at 2 h, followed by a decrease at 4 h. Exposure to LPS and poly-L-arginine led to a significant 7- to 8-fold increase of IL-6 and IL-8 mRNA at 2 h, suggestive of an enhanced transcriptional activity. Subsequently, IL-6 and IL-8 mRNA levels decreased and reached a plateau at 6 h, 2 to 3 times higher than LPS alone (Figures 6A and 6B). TNF-α induced IL-6 and IL-8 mRNA 2- to 3-fold at 1 h in NCI-H292 cells in MEM-A^+^. In the absence of arginine, basal IL-6 and IL-8 mRNA levels are enhanced, in line with the enhanced basal production of IL-6 and IL-8. These enhanced mRNA levels, however, at large run parallel to those for cells in the presence of arginine, suggestive of no enhanced transcriptional activity. The peak for IL-6 mRNA (Figure 6C) appeared slightly broader, suggestive of an attenuated IL-6 mRNA degradation. At 24 h, the IL-6 mRNA levels increased, but this varied from one to another experiment (Figure 6C).

**Figure 7:**
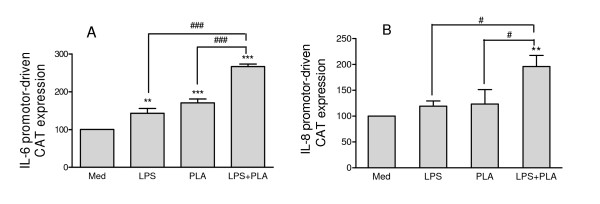
**Modulation of IL-6 (A) and IL-8 (B) promoter-driven transcription by LPS and poly-L-arginine**. Cells were transfected with an IL-6 or IL-8 promoter-driven chloramphenicol acetyltransferase (CAT)-expressing vector. Transfected cells were exposed to normal medium (Med), medium containing 5 μg/ml lipopolysaccharide (LPS), medium containing 5 μg/ml poly-L-arginine (PLA) or medium containing LPS plus PLA (LPS+PLA) for 18 h, respectively. The production of CAT was measured by ELISA. Mean ± SD of three experiments are shown. **P < 0.01 and ***P < 0.001 as compared to Med, ^#^P < 0.05 and ^###^P < 0.001 as compared to LPS+PLA.

The mRNA profiles with poly-L-arginine are indicative of an enhanced transcriptional activity, and thus we transfected NCI-H292 cells with 5 μg of chloramphenicol acetyltransferase (CAT) reporter constructs driven by the wild-type IL-6 or IL-8 promoter. Poly-L-arginine induced IL-6 and IL-8 gene transcription as deduced by quantification of CAT (Figure 7). In the absence of arginine, there was no further enhanced transcriptional activity as determined with CAT reporter constructs (data not shown). Previously we have shown that the hyperresponsive IL-6 and IL-8 production in the absence of tryptophan was due to a reduced IL-6 and IL-8 mRNA degradation [9]. IL-6 and IL-8 mRNA degradation at low arginine levels and 2 hrs after stimulation, using actinomycin D to block transcription, however, did not reveal a reduced mRNA degradation.

## Discussion

The catabolism of arginine in airways of patients with asthma is enhanced and may lead to reduced bioavailability of arginine. Here we show that the reduced arginine levels per se may contribute to inflammation by mediating an exaggerated epithelial production of pro-inflammatory mediators. We anticipated that polycationic proteins, that are abundantly present in the airways of patients with asthma and that have been shown to attenuate cellular arginine uptake, would also lead to exaggerated IL-6 and IL-8 production. The synthetic polycation poly-L-arginine and major basic protein (MBP) triggered an exaggerated IL-6 and IL-8 production, but this appeared not related to reduced arginine uptake.

The exaggerated IL-6 and IL-8 production by NCI-H292 airway epithelial cells due to arginine deficiency was ablated at arginine concentrations above 20 μM. The arginine concentration in airways of patients with asthma is not known, and when known, this concentration may deviate from that of the micro-milieu surrounding the airway epithelial cells. Enzymes expressed by airway epithelial cells of patients with asthma and that catabolise arginine are arginase I and II (EC 3.5.3.1) and the inducible nitric-oxide synthases (iNOS; EC 1.14.13.39). The K_m _values for arginase I, II and nitric-oxide synthase at physiological pH are 0.08 mM, 4.8 mM and 0.0044 mM, respectively [27]. iNOS gives rise to both superoxide and NO at suboptimal levels of arginine, yielding the potent oxidant peroxynitrite [11,24,25] that promotes tissue damage and thus inflammation [26]. As peroxynitrite-derived nitrotyrosines are found in exhaled breath condensates of patients with asthma [28] this suggests that arginine concentrations in airways of asthmatics are below 4.4 μM. In a study by Heinzel and coworkers [25] the brain nitric-oxide synthase was found to produce hydrogen peroxide (as a measure of superoxide) maximally at 1 μM arginine and lower. Taken together this indicates that the concentration range of arginine that gives rise to exaggerated IL-6 and IL-8 responses by airway epithelial cells may occur at pathophysiological conditions. In our experimental set up NOS activity did not contribute to the exaggerated IL-6 and IL-8 responses. In vivo, however, iNOS activity may be present and give rise to peroxynitrites that further aggravate inflammation.

Although we did not primarily aim to clarify the underlying mechanism for the exaggerated IL-6 and IL-8 production, the mRNA profile and the lack of a further increased transcriptional activity suggest that the low arginine concentrations affect the post-transcriptional regulation of IL-6 and IL-8 production. Similar findings were obtained earlier for airway epithelial cells exposed to reduced levels of the essential amino acid tryptophan [9]. At reduced tryptophan levels, IL-6 and IL-8 mRNA degradation was attenuated, resulting in markedly enhanced IL-6 and IL-8 mRNA levels (i.e. superinduction). Despite the reduced protein synthesis capacity of cells, these enhanced levels of IL-6 and IL-8 mRNA apparently outcompete other mRNAs for the remaining translational activity, giving rise to exaggerated IL-6 and IL-8 production. Our findings with reduced arginine concentrations did not convincingly show a reduced IL-6 and IL-8 mRNA degradation. This may be due to the fact that arginine is also produced by the cells themselves and thus the effect on protein synthesis and therefore on IL-6 and IL-8 mRNA degradation may be less than for tryptophan. This is corroborated by the relatively small increase in IL-6 and IL-8 mRNA levels compared to that for control cells and in comparison to that in the absence of tryptophan [9]. Alternatively, we can not as yet exclude the possibility that the IL-6 and IL-8 mRNA degradation is affected at time points beyond that we have assessed now.

Polycationic proteins, such as those derived from degranulating eosinophils, cause bronchial hyperresponsiveness and airway inflammation in experimental animal models upon introduction of the airways [10,29,30]. Although these polycationic proteins are cytotoxic, at lower doses they inhibit the uptake of arginine via the cationic amino acid transporters (CAT; [15]). This reduced uptake of arginine was shown to limit NO production which was found to underlie the bronchial hyperresponsiveness [29-31]. The polycationic peptide poly-L-arginine inhibits arginine uptake more effectively than other cationic proteins [32], as indeed was confirmed here for MBP and epithelial cells. Poly-L-arginine and MBP potently induced IL-6 and IL-8 production by epithelial cells, but this did not coincide with maximal inhibition of arginine uptake, suggesting that it is independent of blocking arginine uptake. In addition, poly-L-arginine promoted transcriptional activity which was not seen for reduced arginine levels. So, we propose that the exaggerated IL-6 and IL-8 production by polycationic proteins is not due to reduced arginine availability. Interestingly, this synergism on inflammatory mediator production has been described before for LPS-stimulated human whole blood with poly-L-arginine [33]. As poly-L-arginine binds to CD14 [12] and since a close interaction between CD14 and TLR4 is required for adequate LPS stimulation [34] it may be argued that poly-L-arginine promotes the physical interaction of CD14 and TLR4 giving rise to NFκB activation [35,36]. As NHBE cells are relatively unresponsive to LPS [37] it is not surprising that we found no synergism between poly-L-arginine and LPS in the IL-6 and IL-8 production by NHBE.

For experimental reasons (see Material and Methods), the arginine uptake experiments were performed at 10 μM arginine and the experiments for IL-6 and IL-8 production at about 1 mM arginine. Since Hammermann et al. (Figure 5 in [15]) showed that inhibition of arginine uptake by poly-L-arginine is similar at 0 and 100 μM arginine in the medium, it is likely that poly-L-arginine also inhibited arginine uptake markedly at 1 mM arginine. As yet we can not exclude that reduced arginine uptake also contributed to the poly-L-arginine-induced exaggerated IL-6 and IL-8 responses. Interestingly we showed that TNF-α in the presence of poly-L-arginine synergized in IL-6 and IL-8 production by NHBE cells. Previously, Visigalli *et al*. [38] found that human endothelial cells exposed to TNF-α stimulate arginine uptake via NF-κB. So, if TNF-α promotes arginine uptake by NHBE cells, poly-L-arginine may counteract and cause a relative arginine deficiency, leading to enhanced IL-6 and IL-8 production. Alternatively, we can not exclude that the prolonged incubation with poly-L-arginine, as is manifest during the 20 h incubation for cytokine production, culminates in a more profound inhibition of arginine uptake and consequently enhances IL-6 and IL-8 production as shown for reduced arginine concentrations.

Comparison of the current findings for both NCI-H292 and NHBE cells reveals some important similarities as well as differences. Both cell types display exaggerated IL-6 and IL-8 responses in arginine-deficient media in the absence of stimuli and in the presence of TNF-α, indicating that this is a general feature of airway epithelial cells, at least in vitro. LPS failed to induce exaggerated IL-6 and IL-8 responses in arginine-deficient media, but this is due to the absence of FCS (see Figure 1). In FCS, soluble CD14 and LPS-binding protein are present which promote binding of LPS to cells and enhance production of mediators [39]. This does not exclude an alternative explanation, i.e. that NHBE cells, at least in vitro, are less responsive to LPS.

The findings for poly-L-arginine appear quite different between NCI-H292 and NHBE cells. Whereas poly-L-arginine and MBP partially inhibit arginine uptake by NCI-H292 cells, we found no effect of poly-L-arginine on arginine uptake by NHBE cells. NCI-H292 cells are tumor-derived cells which typically display an enhanced metabolic activity, and thus these cells may have a higher need for extracellular arginine, as opposed to NHBE cells, making the NCI-H292 cells more vulnerable to inhibitors of arginine uptake. Despite this difference, both NCI-H292 and NHBE cells exposed to poly-L-arginine display exaggerated IL-6 and IL-8 responses in the presence of poly-L-arginine, albeit in response to different stimuli. Our findings for LPS-stimulated NCI-H292 cells in the presence of poly-L-arginine indicate that poly-L-arginine synergizes with LPS (Figure 4) leading to an enhanced transcriptional activity (Figures 6 and 7). Poly-L-arginine may bind to the overall negatively charged cellular membranes and in this way trigger gene transcription. The different responses of NHBE and NCI-H292 cells to LPS in the presence of poly-L-arginine may relate to the low responsiveness of NHBE cells to LPS in vitro.

In the current study we have focussed on IL-6 and IL-8, both key immune-regulatory mediators. IL-6 and IL-8 are encoded by labile mRNAs, which is a common feature of mRNAs encoding mediators such VEGF and IP-10. Therefore the current results for IL-6 and IL-8 responses may apply as well to responses of other mediators that are produced by epithelial cells.

Whether these exaggerated IL-6 and IL-8 responses due to reduced arginine bioavailability and/or reduced uptake of arginine are manifest in vivo remains to be tested. Two recent experimental animal studies addressed the role of arginase in allergic inflammation by inhibiting arginase activity; one showing attenuation of inflammation [40], whereas the other [41] showed an increase. Arginine bioavailability depends not only on arginase activity and involves amongst others iNOS activity and the presence of polycationic proteins, which may contribute to the opposite findings with the two animal studies. In the current study we have tested the effect of poly-L-arginine on IL-6 and IL-8 production in the presence of about 1 mM arginine. Poly-L-arginine, however, may reduce arginine bioavailability more profoundly at conditions with reduced extracellular amounts of arginine and/or an enhanced requirement for intracellular arginine. Such conditions may occur in patients with asthma, due to enhanced arginase and iNOS activities by airway epithelial cells. Therefore, polycationic proteins in the airways of asthmatics may give rise to exaggerated IL-6 and IL-8 responses by both the arginine uptake independent and dependent mechanisms.

## Conclusion

In conclusion, reduced bioavailability of arginine in the airways may enhance inflammation by enhancing pro-inflammatory mediator production by epithelial cells. As pro-inflammatory peroxynitrites can also be produced at low arginine availability, low local arginine concentrations may be an important denominator in controlling airway inflammation. Polycationic proteins/peptides also enhance the epithelial pro-inflammatory mediator production but this is not primarily regulated through reduced arginine concentrations.

## Abbreviations

CAT: chloramphenicol acetyltransferase; FCS: fetal calf serum; HBSS: Hanks' balanced salt solution; iNOS: inducible nitric oxide synthase; L-NAME: N-Nitro arginine methyl ester; MBP: major basic protein; MEM: minimum essential medium Eagle; NHBE: normal human bronchial epithelial cells; NO: nitric oxide; PBS: phosphate-buffered saline.

## Competing interests

The authors declare that they have no competing interests.

## Authors' contributions

X-YF carried out most in vitro experiments, helped to draft the manuscript and performed the statistical analyses. AvdB and MS supervised and performed the molecular biology experiments, whereas LGvdF and BS carried out the initial studies which have led to the present study. HMJ and R-YL helped to draft the manuscript. RL conceived the study, participated in its design and coordination and drafted the manuscript. All authors have read and approved the final manuscript.

## References

[B1] Busse WW, Lemanske RF (2001). Asthma. N Engl J Med.

[B2] Elias JA, Lee CG, Zheng T, Ma B, Homer RJ, Zhu Z (2003). New insights into the pathogenesis of asthma. J Clin Invest.

[B3] Fei GH, Liu RY, Zhang ZH, Zhou JN (2004). Alterations in circadian rhythms of melatonin and cortisol in patients with bronchial asthma. Acta Pharmacol Sin.

[B4] Yates DH, Kharitonov SA, Thomas PS, Barnes PJ (1996). Endogenous nitric oxide is decreased in asthmatic patients by an inhibitor of inducible nitric oxide synthase. Am J Respir Crit Care Med.

[B5] Kharitonov SA, O'Connor BJ, Evans DJ, Barnes PJ (1995). Allergen-induced late asthmatic reactions are associated with elevation of exhaled nitric oxide. Am J Respir Crit Care Med.

[B6] Kochañski L, Kossmann S, Rogala E, Dwornicki J (1980). Sputum arginase activity in bronchial asthma. Pneumonol Pol.

[B7] Zimmermann N, King NE, Laporte J, Yang M, Mishra A, Pope SM, Muntel EE, Witte DP, Pegg AA, Foster PS, Hamid Q, Rothenberg ME (2003). Dissection of experimental asthma with DNA microarray analysis identifies arginase in asthma pathogenesis. J Clin Invest.

[B8] Morris CR, Poljakovic M, Lavrisha L, Machado L, Kuypers FA, Morris SM (2004). Decreased arginine bioavailability and increased serum arginase activity in asthma. Am J Respir Crit Care Med.

[B9] van Wissen M, Snoek M, Smids B, Jansen HM, Lutter R (2002). IFN-gamma amplifies IL-6 and IL-8 responses by airway epithelial-like cells via indoleamine 2,3-dioxygenase. J Immunol.

[B10] Arseneault D, Maghni K, Sirois P (1999). Selective inflammatory response induced by intratracheal and intravenous administration of poly-L-arginine in guinea pig lungs. Inflammation.

[B11] Beckman JS, Koppenol WH (1996). Nitric oxide, superoxide, and peroxynitrite: the good, the bad, and ugly. Am J Physiol.

[B12] Bosshart H, Heinzelmann M (2002). arginine-Rich Cationic Polypeptides Amplify Lipopolysaccharide-Induced Monocyte Activation. Infect Immun.

[B13] Closs EI, Simon A, Vekony N, Rotmann A (2004). Plasma membrane transporters for arginine. J Nutr.

[B14] Closs EI, Boissel JP, Habermeier A, Rotmann A (2006). Structure and function of cationic amino acid transporters (CATs). J Membr Biol.

[B15] Hammermann R, Hirschmann J, Hey C, Mossner J, Folkerts G, Nijkamp FP, Wessler I, Racke K (1999). Cationic proteins inhibit arginine uptake in rat alveolar macrophages and tracheal epithelial cells. Implications for nitric oxide synthesis. Am J Respir Cell Mol Biol.

[B16] Helle M, Boeije L, de Groot E, de Vos A, Aarden L (1991). Sensitive ELISA for interleukin-6. Detection of IL-6 in biological fluids: synovial fluids and sera. J Immunol Methods.

[B17] Hack CE, Hart M, van Schijndel RJ, Eerenberg AJ, Nuijens JH, Thijs LG, Aarden LA (1992). Interleukin-8 in sepsis: relation to shock and inflammatory mediators. Infect Immun.

[B18] Roger T, Out T, Mukaida N, Matsushima K, Jansen H, Lutter R (1998). Enhanced AP-1 and NF-kappaB activities and stability of interleukin 8 (IL-8) transcripts are implicated in IL-8 mRNA superinduction in lung epithelial H292 cells. Biochem J.

[B19] Roger T, Out TA, Jansen HM, Lutter R (1998). Superinduction of interleukin-6 mRNA in lung epithelial H292 cells depends on transiently increased C/EBP activity and durable increased mRNA stability. Biochim Biophys Acta.

[B20] Roger T, Bresser P, Snoek M, Sluijs K van der, Berg A van den, Nijhuis M, Jansen HM, Lutter R (2004). Exaggerated IL-8 and IL-6 responses to TNF-alpha by parainfluenza virus type 4-infected NCI-H292 cells. Am J Physiol Lung Cell Mol Physiol.

[B21] Berg A van den, Kuiper M, Snoek M, Timens W, Postma DS, Jansen HM, Lutter R (2005). Interleukin-17 induces hyperresponsive interleukin-8 and interleukin-6 production to tumor necrosis factor-alpha in structural lung cells. Am J Respir Cell Mol Biol.

[B22] Mukaida N, Mahe Y, Matsushima K (1990). interaction of nuclear factor-kappa B- and cis-regulatory enhancer binding protein-like factor binding elements in activating the interleukin-8 gene by pro-inflammatory cytokines. J Biol Chem.

[B23] Libermann TA, Baltimore D (1990). Activation of interleukin-6 gene expression through the NF-kappa B transcription factor. Mol Cell Biol.

[B24] Meurs H, Maarsingh H, Zaagsma J (2003). Arginase and asthma: novel insights into nitric oxide homeostasis and airway hyperresponsiveness. Trends Pharmacol Sci.

[B25] Heinzel B, John M, Klatt P, Böhme E, Mayer B (1992). Ca2+/calmodulin-dependent formation of hydrogen peroxide by brain nitric oxide synthase. Biochem J.

[B26] Muijsers RB, Veeken A van der, Habernickel J, Folkerts G, Postma DS, Nijkamp FP (2002). Intra-luminal exposure of murine airways to peroxynitrite causes inflammation but not hyperresponsiveness. Inflamm Res.

[B27] Di Costanzo L, Sabio G, Mora A, Rodriguez PC, Ochoa AC, Centeno F, Christianson DW (2005). Crystal structure of human arginase I at 1.29-A resolution and exploration of inhibition in the immune response. Proc Natl Acad Sci USA.

[B28] Hanazawa T, Kharitonov SA, Barnes PJ (2000). Increased nitrotyrosine in exhaled breath condensate of patients with asthma. Am J Respir Crit Care Med.

[B29] Coyle AJ, Ackerman SJ, Burch R, Proud D, Irvin CG (1995). Human eosinophil-granule major basic protein and synthetic polycations induce airway hyperresponsiveness in vivo dependent on bradykinin generation. J Clin Invest.

[B30] Coyle AJ, Uchida D, Ackerman SJ, Mitzner W, Irvin CG (1994). Role of cationic proteins in the airway. Hyperresponsiveness due to airway inflammation. Am J Respir Crit Care Med.

[B31] Meurs H, Schuurman FE, Duyvendak M, Zaagsma J (1999). Deficiency of nitric oxide in polycation-induced airway hyperreactivity. Br J Pharmacol.

[B32] Jarman ER, Lamb JR (2004). Reversal of established CD4+ type 2 T helper-mediated allergic airway inflammation and eosinophilia by therapeutic treatment with DNA vaccines limits progression towards chronic inflammation and remodeling. Immunology.

[B33] Bosshart H, Heinzelmann M (2003). Endotoxin-neutralizing effects of histidine-rich peptides. FEBS.

[B34] Jiang Q, Akashi S, Miyake K, Petty HR (2000). Lipopolysaccharide induces physical proximity between CD14 and toll-like receptor 4 (TLR4) prior to nuclear translocation of NF-kappa B. J Immunol.

[B35] Furuta GT, Ackerman SJ, Varga J, Spiess AM, Wang MY, Wershil BK (2000). Eosinophil granule-derived major basic protein induces IL-8 expression in human intestinal myofibroblasts. Clin Exp Immunol.

[B36] Page SM, Gleich GJ, Roebuck KA, Thomas LL (1999). Stimulation of Neutrophil Interleukin-8 Production by Eosinophil Granule Major Basic Protein. Am J Respir Cell Mol Biol.

[B37] Becker S, Quay J, Koren HS, Haskill JS (1994). Constitutive and stimulated MCP-1, GRO alpha, beta, and gamma expression in human airway epithelium and bronchoalveolar macrophages. Am J Physiol.

[B38] Visigalli R, Bussolati O, Sala R, Barilli A, Rotoli BM, Parolari A, Alamanni F, Gazzola GC, Dall'Asta V (2004). The stimulation of arginine transport by TNFalpha in human endothelial cells depends on NF-kappaB activation. Biochim Biophys Acta.

[B39] Heumann D, Gallay P, Barras C, Zaech P, Ulevitch RJ, Tobias PS, Glauser MP, Baumgartner JD (1992). Control of lipopolysaccharide (LPS) binding and LPS-induced tumor necrosis factor secretion in human peripheral blood monocytes. J Immunol.

[B40] Maarsingh H, Zuidhof AB, Bos IS, van Duin M, Boucher JL, Zaagsma J, Meurs H (2008). Arginase inhibition protects against allergen-induced airway obstruction, hyperresponsiveness, and inflammation. Am J Respir Crit Care Med.

[B41] Ckless K, Lampert A, Reiss J, Kasahara D, Poynter ME, Irvin CG, Lundblad LK, Norton R, Vliet A van der, Janssen-Heininger YM (2008). Inhibition of arginase activity enhances inflammation in mice with allergic airway disease, in association with increases in protein S-nitrosylation and tyrosine nitration. J Immunol.

